# TERT translocation to mitochondria: Exploring its role in mitochondrial homeostasis

**DOI:** 10.1371/journal.pgen.1011923

**Published:** 2025-10-27

**Authors:** Jessica Marinaccio, Erica Rossi, Emanuela Micheli, Ion Udroiu, Nicolò Baranzini, Gaia Marcolli, Antonella Sgura

**Affiliations:** 1 Department of Science, Roma Tre University, Rome, Italy; 2 Department of Biotechnology and Life Sciences, University of Insubria, Varese, Italy; Hebrew University of Jerusalem Faculty of Science, ISRAEL

## Abstract

Telomerase Reverse Transcriptase (TERT), in addition to its well-known role in telomere lengthening, also has non-canonical functions, including gene regulation and protection against apoptosis. Beyond its nuclear functions, it is now recognized for its presence inside mitochondria. However, the biological role of TERT in mitochondrial physiological activity, with its specific mechanism of action, still needs to be clarified. This work clearly demonstrates the presence of TERT inside the mitochondrion under physiological conditions, in different cellular contexts, both with endogenous and ectopic TERT expression, and regardless of the presence of telomerase RNA counterpart TERC. TERT was shown to bind mitochondrial DNA, influencing mitochondrial replication and transcription. Furthermore, electron microscopy analysis of morphology revealed TERT-induced fragmentation of the mitochondrial network. Collectively, our findings suggest that TERT may play a role in regulating mitochondrial biogenesis and dynamics, and influencing processes such as fission and mitophagy, essential for maintaining mitochondrial homeostasis and closely connected to cellular states.

## Author summary

Aside from its main function in the nucleus, telomerase reverse transcriptase (TERT) has been found to have non-canonical roles in different pathways. Here, we demonstrated the presence of TERT inside the mitochondrion in different cellular contexts, both in tumor and normal cell lines and even under physiological conditions, differently from previous studies. We found that TERT associates with mtDNA, influencing mitochondrial replication and transcription and that it affects some mitochondrial features, such as mass, dimension and respiration. Based on the results reported in this study, we suggest that TERT regulates mitochondrial morphology and biogenesis, impacting mitochondrial homeostasis and cellular states. Given the mitochondrial involvement in oxidative stress regulation and apoptosis, the non-canonical TERT roles as antioxidant and anti-apoptotic factor appear to be mitochondria-mediated functions. Moreover, since the mitochondrion is considered a mediator of tumorigenesis and TERT is expressed in 85% of tumors, clarifying the mitochondrial TERT mechanism of action and its connection with mitochondrial functions will help better define the role of mitochondria in cancer.

## Introduction

Mitochondria are membrane-enclosed organelles, found in the cytoplasm of eukaryotic cells, which carry out various crucial cellular functions, including oxidative phosphorylation, central carbon metabolism, and the biosynthesis of intermediates required for cell growth. Moreover, they are responsible for several other essential processes that determine cell function and fate, and alteration of their homeostasis is linked to aging, cancer and various neurodegenerative diseases [1].

The human mitochondrial proteome is much bigger than expected containing around 1100 proteins [2]: this number significantly exceeds the 13 proteins codified by the mitochondrial DNA and all the proteins required for the well-established mitochondrial functions. Indeed, several proteins known for their activity in other cellular compartments are found to localize in mitochondria, with a function that still requires to be clarified and TERT is one of these proteins [3–5].

Telomerase reverse transcriptase (TERT) is, by definition, the catalytic subunit of the enzyme responsible for the elongation of telomeric DNA, acting as a reverse transcriptase with the RNA template TERC, which is part of the telomerase complex [6]. However, TERT function is not limited to telomeres, but it has been found to have a role in different pathways, not related to its reverse transcriptase activity [7,8]. Furthermore, in the last two decades, it has been widely explored the role of TERT beside the canonical one in telomere maintenance, as comprehensively summarized by Udroiu et al. [9]. Because of its main activity on telomeric DNA, TERT is expected to localize in the nucleus, but its sequence is characterized by a nuclear exporting signal and a mitochondrial translocation signal, which together confer the ability to migrate in the cytoplasm and subsequently into mitochondria [10]. TERT ability to localize into mitochondria upon oxidative stress has been demonstrated by several studies, showing a protection of mitochondrial functions by reducing ROS levels and mtDNA damage [11–14].

To date, the literature predominantly recognizes an antioxidant and anti-apoptotic role for TERT [15], even if there are few papers in the opposite direction with the evidence of TERT inducing mtDNA damage [10,16]. At the same time, the presence of TERT within mitochondria is now well-established, although its translocation has mostly been linked to the induction of stress conditions. However, it has also been observed in basal conditions, albeit without much attention being drawn to it [13]. Saretzki’s group, demonstrated that a specifically mitochondrial-targeted TERT prevents nuclear DNA damage, thanks to a reduced level of mitochondrial ROS, after exposure to H_2_O_2_ and X-irradiation [13].

Although several studies show a role of TERT in the mitochondrion, neither its effects nor the reason why TERT enters this organelle are yet clear. In this study, we aimed to investigate these two aspects, starting from some evidence found in the literature.

Since one of TERT non-canonical functions in the nucleus comprises regulation of gene expression in different pathways, such as NF-κB [17] and Wnt/β-catenin [18], it is reasonable to hypothesize a similar function in the mitochondrion; in support of this, TERT was shown to be able to bind mtDNA [12,19]. However, it is essential to first examine how the mitochondrion operates, both with regard to its transcription and replication. The regulation of mtDNA expression is quite complex and operates through multiple levels of control [20]. The human mitochondrial genome is a 16.6kb-long sequence and contains 37 genes, lacking introns and encoding 13 proteins involved in oxidative phosphorylation (OXPHOS), two ribosomal RNAs, and 22 transfer RNAs [20,21]. mtDNA is packaged into nucleoid structures by various proteins, with mitochondrial transcription factor A (TFAM) being the primary protein and responsible for mtDNA compaction [22]. Other proteins include those involved in the replication and transcription, among which mitochondrial RNA polymerase (POLRMT), mitochondrial single-stranded DNA-binding protein (mtSSB), mitochondrial polymerase *γ* (POLG), and Twinkle helicase [23]. Nucleoid dynamics represents a pivotal parameter in regulating both mtDNA replication and transcription. In particular, the degree of mtDNA compaction, depending on TFAM level, dictates the accessibility of the mtDNA to replication and transcription complexes.

In this study, we aim to provide a clearer overview of the role of TERT in mitochondria, by comparing different cellular contexts: normal and TERT-overexpressing fibroblasts, tumor cells with both endogenous and exogenous expression of TERT, and cells that either express or lack the telomerase RNA subunit TERC. Based on data previously reported in literature, we hypothesized that TERT is involved in preserving mitochondrial functions even under basal conditions. Therefore, we decided to conduct our experiments in the absence of stress conditions. We indeed demonstrated that when TERT is expressed, it translocates to the mitochondrion, regardless of the presence of TERC in this compartment. Furthermore, we showed that TERT binds to mitochondrial DNA, thereby affecting mtDNA replication and transcription. Considering our findings on mitochondrial morphology, it is intriguing to propose that TERT is involved in regulating mitochondrial dynamics, which is closely linked to mitochondrial functions and cellular states.

## Results

### TERT mitochondrial localization occurs in different cell lines

To determine whether TERT mitochondrial localization depends on a specific cellular context, we examined a panel of different cell lines (Table 1). Unlike previous studies, we conducted our investigation under physiological conditions, without inducing oxidative stress. First, we analysed two telomerase-positive tumors (HCT116 and SK-MEL28), normally expressing TERT and presenting telomerase activity; then, we induced TERT expression in two TERT-lacking cancer cell lines (U2OS and SAOS) (S1A Fig), characterized by the alternative mechanism of telomere lengthening (ALT). In addition, these two ALT-positive cell lines allowed us to compare the condition in presence or absence of the RNA component of telomerase (TERC), essential for the canonical telomerase activity: namely, TERC expression is present in SAOS but absent in U2OS. This feature was confirmed by our RT-qPCR data measuring TERC RNA levels and by RQ-TRAP assay detecting telomerase activity (S1B-S1C Fig): as expected, SAOS displayed TERC expression, even at low level, that is increased when TERT expression is induced (in SAOS^TERT-HA^), likely because TERC is stabilized by TERT binding [24], and its incorporation into the telomerase complex prevents its degradation. Consequently, when TERT is overexpressed in SAOS^TERT-HA^, telomerase activation was observed (S1C Fig). Conversely, U2OS cells displayed neither TERC expression nor telomerase activity, even in the presence of TERT overexpression.

**Table 1:** List of the cell lines used in this study with their characteristics.

Cell line	Type	TERT expression	TERC expression	Telomerase activity
**HFFF2**	Foreskin Fibroblasts	NO	YES	NO
**HFFF2** ^**TERT-HA**^	Foreskin Fibroblasts	YES	YES	YES
**U2OS**	Osteosarcoma	NO	NO	NO
**U2OS** ^**TERT-HA**^	Osteosarcoma	YES	NO	NO
**SAOS**	Osteosarcoma	NO	YES	NO
**SAOS** ^**TERT-HA**^	Osteosarcoma	YES	YES	YES
**SK-MEL28**	Melanoma	YES	YES	YES
**HCT116**	Colon Adenocarcinoma	YES	YES	YES

**Fig 1:**
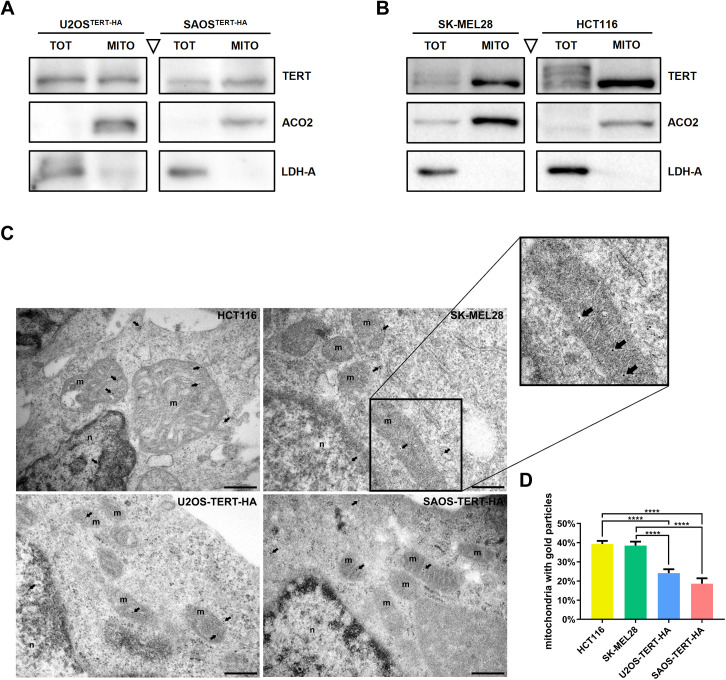
TERT presence in mitochondria of different cell lines. (A-B) Western blot analysis of TERT expression in whole cell lysate (TOT) and mitochondrial lysate (MITO), from the TERT-HA transduced cell lines U2OS^TERT-HA^ and SAOS^TERT-HA^ in (A) and from telomerase positive cells SK-MEL28 and HCT116 in (B). Lactate dehydrogenase A (LDH-A) and Aconitase 2 (ACO2) were used as cytosolic and mitochondrial control protein, respectively. (C) TEM representative micrographs of TERT immunogold labeling in HCT116, SK-MEL28, U2OS^TERT-HA^ and SAOS^TERT-HA^ cell lines. Mitochondria (m), nucleus (n) and TERT gold particles in mitochondria (black arrows) are indicated. Scale bar: 300 nm. (D) Percentage of mitochondria containing gold particles. Data expressed as mean values ± SEM. Statistical analysis is performed with respect to HCT116 and SK-MEL28 cell lines. **** p < 0.0001, by One-way ANOVA followed by post-hoc Dunnet’s test.

Western blots in Fig 1A and 1B show that TERT is found in the mitochondrial fraction of all the TERT-expressing cell lines (both endogenous and induced), proving that its mitochondrial localization occurs under physiological conditions, and it is independent of TERC expression.

Immunogold labelling in transmission electron microscopy (TEM) enabled us to precisely and directly visualize TERT localization in different cellular compartments, providing clear evidence of its presence within the mitochondria of all the studied cell lines, specifically in the mitochondrial matrix (Figs 1C and S2A). The number of mitochondria per cell and the number of gold particles visualised inside each mitochondrion were counted to determine the percentage of mitochondria containing gold particles (Fig 1D): telomerase-positive cell lines (HCT116 and SK-MEL28) showed a higher percentage of TERT-containing mitochondria than TERT-overexpressing cells.

### TERT translocation in the mitochondrial matrix is independent of TERC

**Fig 2:**
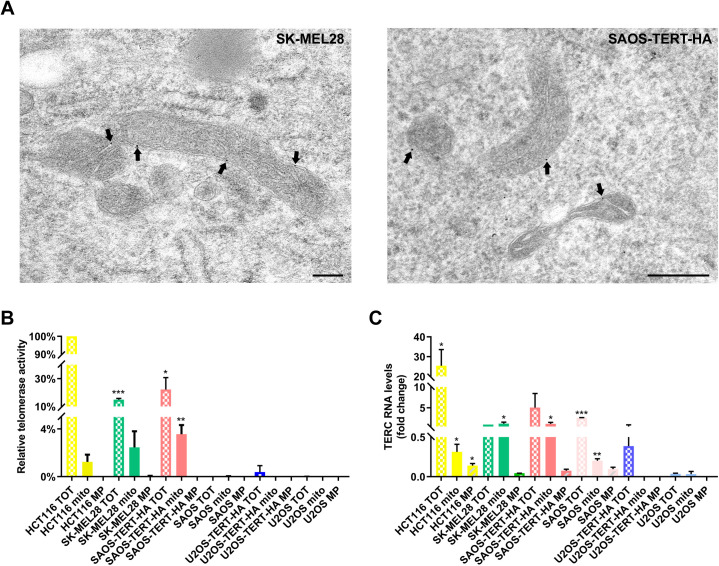
TERC localizes into mitochondrial intermembrane space. (A) EM-ISH with biotin-labeled probe for TERC RNA and two-step immunogold detection (monoclonal rabbit anti-biotin primary antibody and secondary mouse anti-rabbit gold conjugate antibody). Representative micrographs of cell lines with endogenous (SK-MEL28) or ectopic TERT expression (SAOS^TERT-HA^), showing that TERT gold particles (black arrows) were restricted to mitochondrial intermembrane space. Scale bar: 200 nm. (B) RQ-TRAP assay of total lysate (TOT), whole mitochondrial lysate (mito) and mitoplast lysate (MP). Telomerase activity is expressed as percentage relative to HCT116 whole cell lysate sample. Values are presented as mean ± SEM. Statistical analysis is performed using unpaired Student’s t-test, comparing each cell line to U2OS cells, which represent the negative control. (C) Levels of TERC RNA in whole cells (TOT), mitochondria (mito) and mitoplasts (MP) evaluated by RT-qPCR. Values are expressed relative to total RNA of SK-MEL28 as mean ± SEM. Statistical analysis is performed using unpaired Student’s t-test, comparing each cell line to U2OS cells. *p < 0.05,**p < 0.01, ***p < 0.001.

TERT is generally known to act together with the RNA counterpart TERC, that is essential for telomerase canonical activity. Wang’s research group discovered that TERC is imported into mitochondria, where it is processed into a shorter form, named TERC-53, that migrates to the cytoplasm [25,26]; however, it remains to be clarified if TERC exerts any function in the mitochondria. For this reason, it is interesting to understand whether TERT mitochondrial translocation and its consequent function are related to the presence of TERC. We adopted a powerful technique, based on electron microscopy coupled to in-situ hybridization (EM-ISH) [27], that allows to identify and specifically localize this RNA within the mitochondria. We confirmed the presence of TERC in the mitochondria, and a detailed analysis indicated a limited localization within the mitochondrial intermembrane space, as shown by the representative TEM micrographs (Figs 2A and S2B-S2C).

This observation was confirmed by analysis on mitoplast, i.e., digested mitochondrion that preserves the inner membrane with the enclosed matrix, hence removing the outer mitochondrial membrane and the intermembrane space. In the cell lines with expression of both TERC and TERT (HCT116, SK-MEL28, SAOS^TERT-HA^), telomerase activity was observed for the whole mitochondrial lysates (mito) but not when proteins are obtained from mitoplasts (MP) (Fig 2B). In the RT-qPCR analysis on TERC-expressing cell lines (HCT116, SK-MEL28, SAOS and SAOS^TERT-HA^), TERC was detected in the RNA extracted from the whole mitochondrion (mito) but seems to be either absent or present at very low levels, in the mitoplast (MP) (Fig 2C). These results are indicative of TERC residence outside the inner mitochondrial membrane and, since TERT was shown to reside mainly in the mitochondrial matrix [12,19], we can suggest that its function in this compartment is independent of TERC.

### TERT association with mitochondrial DNA and regulation of gene expression

**Fig 3:**
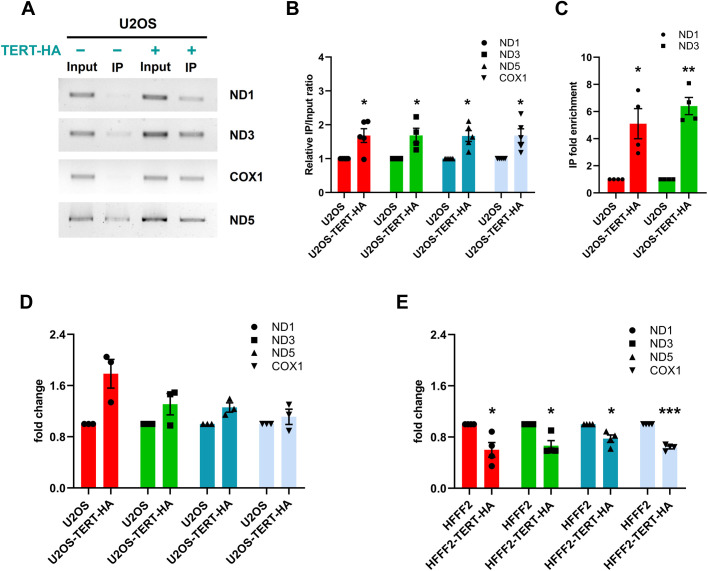
TERT associates with different regions of mtDNA. (A-C) Mitochondrial DNA immunoprecipitation (mIP) performed with anti-HA antibody on whole cell lysates obtained from U2OS^TERT-HA^ and the corresponding control cell line U2OS, after chemical cross-linking. (A) Representative agarose gel electrophoresis of PCR products, obtained from the amplification of different mtDNA regions as indicated (ND1, ND3, ND5 and COX1), on DNA extracted from the lysate before immunoprecipitation (Input) or from the immunoprecipitated sample (IP). (B) Quantification of band intensities, reported as IP/Input ratio. The values are normalized to U2OS cells and expressed as mean ± SEM. (C) Quantitative evaluation by qPCR of ND1 and ND3 genes in the immunoprecipitated samples. The values are normalized to U2OS cells and expressed as mean ± SEM. (D-E) RT-qPCR evaluation of mRNA expression level of ND1, ND3, ND5 and COX1 in U2OS ^TERT-HA^ and HFFF2^TERT-HA^, normalized to U2OS and HFFF2, respectively. Values are expressed as mean ± SEM. Statistical analysis is performed with respect to corresponding control cells. *p < 0.05, **p < 0.01, ***p < 0.001 by paired t-test.

Since it is known that TERT, in the nucleus, is involved in mechanisms other than telomere maintenance, such as gene expression regulation of WNT/β-catenin or NF-kB pathways [17,18], we have hypothesized that it could have a similar function in mitochondria, by interacting with mitochondrial DNA or influencing the binding of mitochondrial transcription factors. For this reason, to determine if TERT binds directly or indirectly to mtDNA, we performed mitochondrial DNA immunoprecipitation (mIP) on U2OS cells overexpressing HA-tagged TERT (U2OS^TERT-HA^) (Figs 3A and S3). We found that TERT is able to associate with the four analysed mtDNA regions (ND1, ND3, ND5 and COXI), that were chosen on purpose scattered along the circular mtDNA sequence; PCR amplification with the specific primers was performed on the DNA extracted from the immunoprecipitated samples (IP), showing the specific products enriched in IP of U2OS^TERT-HA^, compared to IP of U2OS (Fig 3B). To confirm the data with a quantitative method, we also performed qPCR amplification of ND1 and ND3 genes on IP DNA, detecting an enrichment of both genes in U2OS^TERT-HA^ (Fig 3C). This result indicates that TERT associates with different mtDNA regions, probably without a sequence specificity, confirming what previously found [19]. Therefore, TERT function could be not limited to specific regions of the mitochondrial genome, exerting a more extensive role in regulating processes like replication and transcription.

To study the possible effect of this association on mitochondrial gene transcription, we measured by RT-qPCR the mRNA level of the genes found in the immunoprecipitated samples (ND1, ND3, ND5 and COX1): U2OS^TERT-HA^ cells showed not statistically significant increase of transcription compared to U2OS (Fig 3D). Then, we decided to extend our investigation on mtDNA transcription to normal primary fibroblasts (HFFF2) with ectopic TERT overexpression (HFFF2^TERT-HA^). Differently from U2OS^TERT-HA^, we found significantly reduced mRNA levels of the four analysed genes (ND1, ND3, ND5 and COX1) in HFFF2^TERT-HA^ compared to HFFF2 (Fig 3E).

### TERT influence on mtDNA replication and copy number

**Fig 4:**
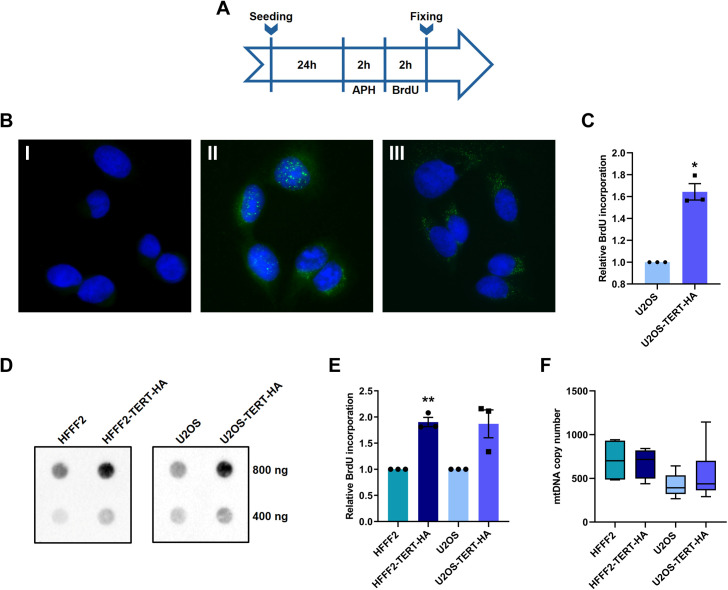
Effects of TERT on mtDNA replication and copy number. (A) Schematic illustration of the working procedure for combined treatment with aphidicolin (APH) and bromodeoxyuridine (BrdU), to induce BrdU incorporation exclusively in the mtDNA. (B) Immunofluorescence with BrdU antibody in U2OS^TERT-HA^ cell lines under the following treatments: (I) only APH for 2 hours; (II) only BrdU for 2 hours; (III) combined APH and BrdU treatment. (C) Evaluation of relative fluorescence corresponding to the amount of incorporated BrdU in mtDNA. The values are normalized to U2OS cells and expressed as mean ± SEM. Statistical analysis is performed with respect to the corresponding control cells, using paired Student’s t-test. (D) Immunoblotting of an equal amount (400 or 800 ng) of DNA extracted from the indicated cell lines, incubated with anti-BrdU antibody. (E) Quantitative analysis of incorporated BrdU levels in mtDNA of HFFF2^TERT-HA^ and U2OS^TERT-HA^, normalized to HFFF2 and U2OS, respectively. Data expressed as mean values ± SEM. Statistical analysis is performed with respect to the corresponding control cells, using paired Student’s t-test. (F) Boxplot of mtDNA copy number determined by qPCR quantification of D-loop mtDNA region. The middle bar represents the median, the box extends from the 25th to the 75th percentiles, and the whiskers indicate the maximum and minimum values. Statistical analysis is performed with respect to each control cell line, using One-way ANOVA. *p < 0.05, **p < 0.01.

Replication and transcription of the mitochondrial genome are known to be closely related, and some regulating proteins are involved in both processes. Hence, we hypothesized that TERT association with mtDNA could alter also mtDNA replication. Taking advantage of a specific inhibitor of nuclear DNA synthesis, aphidicolin, it was possible to selectively monitor mtDNA replication through the thymidine analogue BrdU incorporation. After combined treatment with aphidicolin and BrdU, as schematically described in Fig 4A, cells were visualised by immunofluorescence with anti-BrdU antibody, showing that, in case of the combined treatment, BrdU signal is visible only outside the nucleus (Fig 4B). Evaluation of the relative fluorescence intensity indicates that U2OS^TERT-HA^ cells incorporated higher levels of BrdU compared to U2OS cells (Fig 4C). A similar result was obtained by dot-blotting analysis of DNA isolated from cells after the same treatment. Equal amounts of DNA were spotted on a membrane and revealed by BrdU antibody hybridization: a higher level of BrdU incorporation was detected for the DNA extracted from U2OS^TERT-HA^ compared with DNA from U2OS (Fig 4D-4E), indicating a higher mitochondrial replication rate. To assess that this evidence is related to the presence of TERT and not just due to the high proliferation rate typical of tumor cells, the same experiment was performed on HFFF2^TERT-HA^, leading to a similar result of increased replication, compared to HFFF2.

Considering the increased replication, it is reasonable to expect an elevated mtDNA copy number in U2OS ^TERT-HA^ and HFFF2^TERT-HA^. Total DNA was isolated and the mtDNA/nDNA ratio was determined by RT-qPCR analysis. Surprisingly, the relative mtDNA copy number of HFFF2^TERT-HA^ appeared unaffected and in U2OS^TERT-HA^ it showed a not statistically significant increase but characterized by a large variability (Fig 4F).

### Prediction of TERT protein-protein interactions

**Fig 5:**
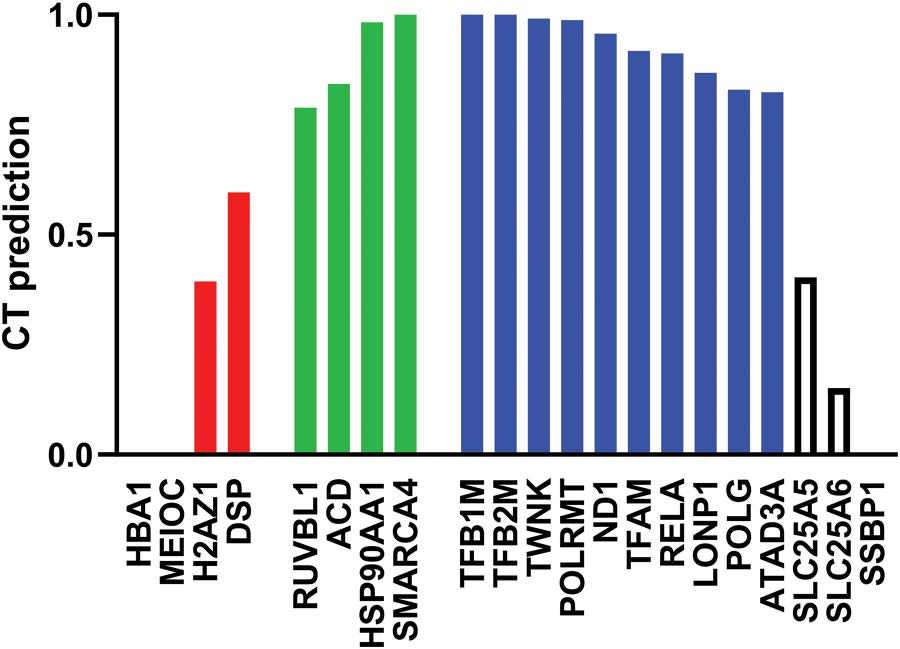
Predictions of interaction with TERT. Values represent the probability of interacting with TERT, predicted by the CT module of PEPPI platform (CT prediction). Negative controls, proteins that are known to not interact with TERT, are shown in red (with values ranging from 0.001 to 0.596), and positive control proteins, known to interact, are shown in green (with values ranging from 0.789 to 1). Proteins with a high probability of interacting with TERT are shown in blue; in white, those with low probabilities.

The observed association of TERT with mtDNA previously described, may suggest either a direct binding to it or a binding mediated through interactions with proteins of the nucleoid complex. This would explain its ability to associate with different mitochondrial genes and affect both replication and transcription. Therefore, we used the neural network-based CT module from the PEPPI platform [28] to predict the probability of interactions between TERT and proteins of the nucleoid complex and/or related to mtDNA replication/transcription (Fig 5). We included as positive controls proteins known to interact with TERT (pontin/RUVBL1, TPP1/ACD, HSP90, SMARCA4) [18,29–31] and as negative controls, protein almost certainly not interacting with it (hemoglobin alpha/HBA1, meiosis-related MEIOC, histone H2AZ1, desmoplakin/DSP). Probability values for positive controls ranged from 0.789 to 1, while values for negative controls were between 0.001 and 0.596. All proteins involved in replication/transcription (TFB1M, TFB2M, TWNK, POLRMT, TFAM, POLG) gave high probability of interacting with TERT (0.83-1). Also, NADH dehydrogenase (ND1), NF-kappa-B p65 subunit (RELA), lon peptidase 1 (LONP1) and mitochondrial ATPase (ATAD3A) gave high probability (>0.82). Other two proteins forming the nucleoid complex, SLC25A5 and SLC25A6 gave values similar to those of negative controls (0.403 and 0.151, respectively). Finally, the single stranded mtDNA binding protein SSBP1 gave a very low probability of interacting with TERT (0.001).

### Modulation of mitochondrial mass and energetic metabolism by TERT overexpression

**Fig 6:**
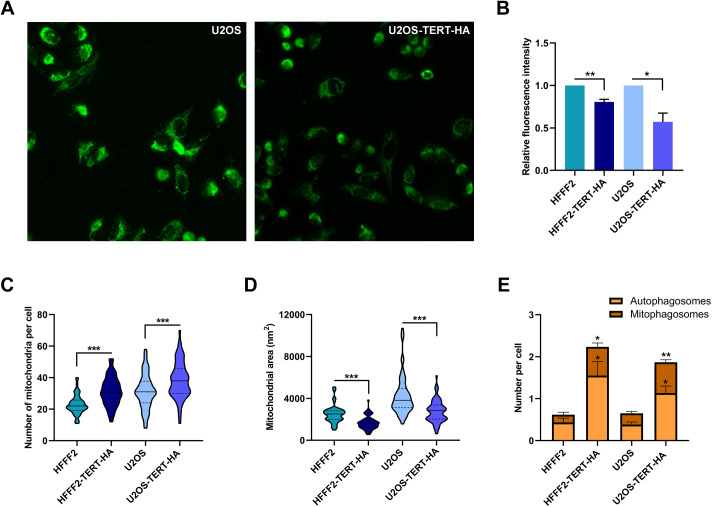
TERT overexpression affects mitochondrial mass and dimension. (A) Representative image of U2OS and U2OS^TERT-HA^ cells stained with MitoTracker Green. (B) Mitochondrial mass reported as relative fluorescence intensity of HFFF2^TERT-HA^ and U2OS^TERT-HA^, with respect to HFFF2 and U2OS respectively. Data expressed as mean values ± SEM. Statistical analysis is performed with respect to the corresponding control cells, using paired Student’s t-test. (C) Number of mitochondria per cell, counted from at least 60 TEM images of a single cell. (D) Total mitochondria cross-section area per single cell measured by TEM imaging. In the violin plot the middle bar represents the median, the box extends from the 25th to the 75th percentile and the whiskers indicate the maximum and minimum values. Statistical analysis is performed comparing each sample to its corresponding control. (E) Frequency of autophagosomes and mitophagosomes events per cell. Data expressed as mean values ± SEM. Statistical analysis is performed independently for autophagosomes and mitophagosomes, with respect to each control cell line, using unpaired Student’s t-test. *p < 0.05; ** p < 0.01; ***p < 0.001.

The observed impact of TERT overexpression on mtDNA replication and transcription prompted us to investigate more broadly the mitochondrial mass and the energetic metabolism. Staining mitochondria with Mitotracker Green (Fig 6A) revealed a decrease in the mitochondrial mass both in HFFF2^TERT-HA^ and U2OS^TERT-HA^, compared to their controls (Fig 6B). Since this evidence appeared to be in contrast with the augmented replication efficiency measured for these cells, we decided to analyse number and area of mitochondria by TEM visualization (Fig 6C-6D and S4A). Both TERT-overexpressing cell lines exhibited a higher number of mitochondria per cell, which were characterized by a smaller area. Even the whole mitochondrial area (as the sum of all the mitochondria within a cell) is reduced in the presence of TERT, providing a possible explanation for the lower mitochondrial mass, detected with Mitotracker Green staining.

A global reduction of mitochondria and an increased replication, observed in TERT-overexpressing cells, were apparently contradictory results, but they might suggest an involvement of TERT in regulation of mitophagy and mitochondrial biogenesis. For this reason, we took advantage of TEM images to count the number of autophagosomes and mitophagosomes (S4B Fig), and we observed a statistically significant higher number of both structures in HFFF2^TERT-HA^ and U2OS^TERT-HA^, compared to their respective controls (Fig 6E).

**Fig 7:**
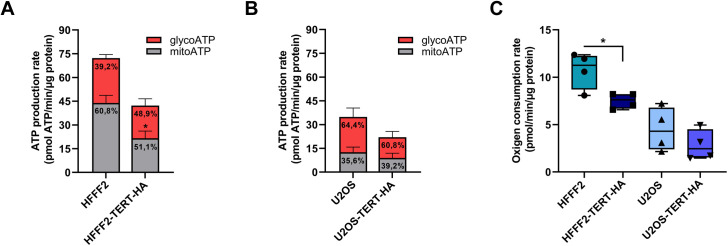
Energetic metabolism is shaped by the cell type and the presence of TERT. (A) Quantification of ATP production measured by Seahorse XF real-time ATP rate assay in HFFF2 and HFFF2^TERT-HA^ and (B) U2OS and U2OS ^TERT-HA^. Data expressed as mean values ± SEM. (C) Boxplot of the basal oxygen consumption rate in HFFF2, U2OS and TERT-HA transduced cells. The middle bar indicates the median, the box extends from the 25th to the 75th percentile and the whiskers show the maximum and minimum values. Statistical analysis is performed compared to HFFF2 or U2OS cells. *p < 0.05 by unpaired Student’s t-test.

To have a glance at the energetic metabolism, we decided to perform the real-time ATP synthesis rate assay on the Seahorse extracellular flux analyser, which measures the total ATP production rate and allows to distinguish between ATP produced from oxidative phosphorylation (mitoATP) and glycolysis (glycoATP) (S5 Fig). It is important to note that U2OS cells, as expected for a tumor cell line, exhibited a predominantly glycolytic metabolism, unlike HFFF2 (Fig 7A). However, the latter cell line, in the presence of TERT, showed a slight metabolic shift, with reduced OXPHOS and increased glycolysis (Fig 7B). In both cell lines, the overexpression of TERT lead to a reduction of the basal oxygen consumption rate (Fig 7C), denoting a lower OXPHOS activity, which could be the consequence of the observed decreased mitochondrial mass.

## Discussion

As mentioned in the introduction, one of the extra-telomeric roles of telomerase reverse transcriptase TERT is performed upon its mitochondrial translocation, where it exerts protective and antioxidant functions. In a previous study, we showed that TERT-overexpressing fibroblasts exhibit lower basal ROS level, reduced induction of oxidative stress (OS) and preserved mitochondria functionality after OS [14]. However, the exact physiological role of TERT within organelles, as well as the specific mechanism of action, remains to be fully understood. In this work, using different techniques, we clearly demonstrated the presence of TERT inside the mitochondrion in different cellular contexts, both with endogenous and ectopic TERT expression. Notably, this was observed under physiological conditions, revealing that TERT mitochondrial translocation occurs even without induction of oxidative stress, although it is known that OS promotes TERT mitochondrial translocation [11,16,32]. TERT canonical function, which involves telomere lengthening, is exerted by the telomerase complex with the RNA TERC. However, our results indicate that the mitochondrial role of TERT is independent of the presence of TERC, even if this RNA is able to migrate to this compartment [25]. Thanks to the comparison between whole mitochondrion and mitoplast (obtained by removing the outer membrane and the intermembrane material), we showed that mitochondrial TERC is restricted to the intermembrane space, whereas TERT localizes in the matrix. This is consistent with previous works indicating that mitochondrial TERT can assemble with other RNAs, distinct from TERC, probably to perform other reverse transcriptase activity [19,33].

Considering that transcription factors, traditionally involved in nuclear gene regulation, have been found to play a role in mitochondrial gene regulation [34], and that a well-known non-telomeric function of TERT is transcriptional regulation [7,8], we suggest that TERT may also perform this role in mitochondria. In this regard, we have carried out mitochondrial immunoprecipitation, showing TERT association with different mtDNA sequences, localized in regions that are well-spaced along the mitochondrial genome; this evidence suggests a lack of sequence specificity and indicates that TERT influence may have a wider effect on the transcription process. Consistently, we observed that TERT overexpression induced a modification of mitochondrial gene expression: mRNA levels were significantly reduced in normal cells (HFFF2^TERT-HA^), while in tumor cells (U2OS^TERT-HA^) the effect was less pronounced, with a tendency toward increased mRNA levels. The reason of this opposite effect could be linked to the different energetic metabolism, which is mainly based on mitochondrial respiration in HFFF2 while on glycolysis in U2OS. In addition, HFFF2^TERT-HA^ displayed a shift towards the glycolytic metabolism, similar to the Warburg effect observed in tumor cells [35], as shown by the analysis of the glyco- and mitoATP production; hence, a reduction in the expression of proteins involved in OXPHOS, such as ND1 and ND3 subunits of complex I, is predictable.

The transcriptional regulation activity may be the outcome of two different regulatory mechanisms, both in agreement with the immunoprecipitation results: either through direct TERT binding on mtDNA sequences or through interaction with other proteins, such as transcription factors or components of the nucleoid complex. Consistent with the latter idea, it was previously shown by Sharma et al. [19] that TERT co-purifies with nucleoid proteins, with TFAM being a highly promising candidate. This is because it is known that the modulation of TFAM levels and its ability to bind mtDNA represent the main mode of transcriptional control [36]. In addition, based on our *in-silico* prediction, TERT was found to have a high probability of interaction with various mitochondrial proteins, particularly those belonging to the nucleoid complex and involved in replication or transcription, such as TFAM, as hypothesized, but also TFB1M, TFB2M, TWNK, POLRMT, and POLG. Alternatively, it is also possible that TERT effects on mitochondria is mediated by interactions with one of some nuclear transcription factors, which were shown to localize to mitochondria and bind mtDNA, influencing its transcription, such as p65 (RelA) subunit of NF-kB [37]. It is interesting to note that, in the nucleus, TERT interacts directly with RelA, increasing its stability and favouring its binding to a subset of NF-κB-dependent promoters [17]. Moreover, this protein was also found among those with a high probability of interaction with TERT in our prediction. Since RelA was shown to regulate mitochondrial gene expression by binding to the D-loop [3], TERT might regulate mtDNA transcription through interaction with p65, possibly modulating its stability or activity on mtDNA. However, this proposed mechanism certainly requires future validation through dedicated experimental evidence.

We should also take into consideration that TERT association with mtDNA could be indicative also of an influence on replication and that transcription may impact replication. Indeed, these two processes are closely interconnected for two reasons: both processes are affected by nucleoid compaction, which regulates mtDNA accessibility, and initiation of replication at the leading-strand origin relies on transcription machinery and the formation of an RNA-DNA hybrid known as an R-loop [38]. Indeed, we observed enhanced mtDNA replication in TERT-overexpressing cells, including both normal primary and tumor cells. This effect could result either from transcriptional regulation or from a direct impact on the replication machinery. An increase in mtDNA replication should be reasonably related to higher mtDNA copy number and mitochondrial mass, but this was not the case. Indeed, we observed highly variable mtDNA copy number without any significant variation and a reduction in mitochondrial mass. This inconsistency led us to propose a model of faster mitochondrial turnover induced by the presence of TERT. We hypothesize that this process occurs through a dual mechanism: on one hand, by fostering mitophagy or other mitochondrial degradation pathways to remove damaged mitochondria, and on the other hand, by stimulating biogenesis to generate healthy newly synthesized mitochondria.

This idea is consistent with the findings of our previous work, which showed that TERT-overexpressing fibroblasts can preserve mitochondrial morphology and functionality after H_2_O_2_-induced oxidative stress, likely due to efficient removal of damaged mitochondria [14]. Based on our findings, we suggest that the observed increase in mitochondrial replication facilitates the renewal of newly synthesized mtDNA, helping to maintain a nearly steady mtDNA copy number.

Another compelling result that could further support this hypothesis is that TERT-overexpressing cells were characterized by a significantly higher number of mitophagosomes and autophagosomes, indicative of an active mitophagy process. Mitophagy refers to a selective sequestration mechanism of damaged or depolarized mitochondria into double-membraned autophagosomes for subsequent lysosomal degradation [39].

Since TERT is known to act as a transcriptional regulator modulating the expression of several genes, such as NF-kB and Wnt [9], it is possible that TERT enhances the expression of a gene involved in a pathway related to mitochondrial biogenesis. Indeed, Ma et al. [40], observed that TERT activates the AMPK/PGC-1α signalling pathway, which regulates mitochondrial homeostasis. PGC-1α, in particular, orchestrates the balance between mitochondrial fusion, fission and mitophagy, and also interact with TFAM [41]. Although this hypothesis can be viewed just as an early suggestion for future research, we wish to stress the need to understand that TERT can affect mitochondrial homeostasis both by its action inside these organelles and by its regulating activity on nuclear genes.

Mitochondria need to be considered as part of a dynamic network of interconnected units, that undergo to continuous process of fission and fusion. Usually, mitochondrial fission and subsequent fragmentation are associated with dysfunctional mitochondria and impaired oxidative phosphorylation, while fused and tubular morphology is ascribable to healthy mitochondrial state and high respiration. However, depending on the cell type, physiological conditions, and changes in bioenergetic requirements, an adaptation mechanism of mitochondrial morphology occurs, that could lead to fragmentation while preserving mitochondrial functionality [42]. We showed that the ectopic expression of TERT resulted in a modification of mitochondrial network, which appeared to be fragmented, as evidenced by a higher number of smaller-sized mitochondria. This evidence reinforces our hypothesis, as a fragmented mitochondrial morphology is indicative of an active fission mechanism, which is generally considered a prerequisite for mitophagy [39,43]. It is likely that in TERT-overexpressing cells, the fission mechanism is constantly activated, ready to respond to an increased oxidative stress or any factors that induce mitochondrial dysfunction or mtDNA damage. This is in agreement with a recent study that reported TERT-induced mitochondrial fission, either in basal or oxidative stress conditions [44].

Interconnected mitochondrial networks are found in metabolically and respiratory active cells, whereas small and fragmented mitochondria characterize quiescent and respiratory inactive cells [42]. In line with this, we observed that the fragmented mitochondrial morphology in U2OS^TERT-HA^ and HFFF2^TERT-HA^ is associated with a lower respiratory capacity, compared to their respective control cell lines. We suggest that mitochondrial fragmentation may not be the reason why OXPHOS is reduced, but rather an adaptation to maintain respiration at a minimal level. TERT-overexpressing cells are characterized by low basal levels of ROS [14], which could be due not only to an increase in the antioxidant system [45], but also to a reduced OXPHOS activity, as we found in this study, which represents the major source of ROS species [46]. Hence, considering that TERT is expressed in 85% of tumor cells, it is intriguing to suggest that reducing the mitochondrial respiration activity might be a strategy implemented by telomerase-positive tumors to minimize ROS production.

TERT contribution to the onset and development of cancer has been usually associated with its main function of telomeric DNA elongation, conferring unlimited proliferative ability to normal cells. Since mitochondrion is considered a mediator of tumorigenesis [47], clarifying the mitochondrial TERT mechanism of action and its connection with mitochondrial functions will help better define the role of mitochondria in cancer.

In the literature, non-canonical TERT roles as antioxidant and anti-apoptotic factor has been reported [15], but how these functions are carried out still needs to be deciphered. The results of this research would provide insights into this topic, likely suggesting that these functions are mitochondria-mediated, given the mitochondrial involvement in oxidative stress regulation and apoptosis.

In conclusion, this study demonstrated that TERT binds mtDNA, influencing mitochondrial replication and transcription and that it is able to affect some mitochondrial features, such as mass, dimension and respiration. Collectively, our results indicate that TERT may not only drive mitochondrial turnover through enhanced replication but also promotes mitophagy and regulates mitochondrial morphology to maintain cellular homeostasis. TERT antioxidant activity appears to be potentially associated with its ability to induce mitochondrial fragmentation, thereby reducing cellular respiration. However, since it is not clear whether all these effects are strictly dependent on its mitochondrial localization or are mediated by a nuclear function, further studies are needed. Nevertheless, a deeper understanding of the mechanism of function of this additional player inside mitochondria could help in better delineating and characterizing the wide panorama of mitochondrial functions.

## Materials and methods

### Cells, culture condition and retroviral transduction

Human Fetal Foreskin Fibroblasts HFFF2 (ECACC, Salisbury, UK), Osteosarcoma cell lines U2OS (ECACC) and Human Epthelial Kidney cells HEK 293 (ECACC) were grown in Dulbecco’s Modified Eagle Medium High Glucose, Osteosarcoma cell lines SaOS-2 (referred to as SAOS throughout the text) (ECACC) in Eagle’s Minimum Essential Medium. Both media were supplemented with 10% fetal bovine serum (Euroclone, Milan, Italy), 10000 units/ml penicillin, 10 mg/ml streptomycin, and 2 mM L-glutamine (Euroclone). Cells were maintained in a humidified incubator at 37°C, with 95% relative humidity and 5% CO_2_.

TERT-HA overexpressing cells were obtained by retroviral transduction as previously reported [14]. Briefly, the vector plasmid pBABE-puro-hTERT-HA (Addgene plasmid #1772), containing the cDNA of hTERT protein with the HA-tag, was transfected into HEK 293 using Lipofectamine 2000 (Thermo Fisher Scientific, Waltham, MA, USA) and Ampho Retrovirus Packaging Vector (Novus Biological, Centennial, CO, USA) to obtain retroviral vector particles. After 72hrs, the supernatant from these cells was used to infect U2OS and SaOS-2 cells to produce respectively the U2OS^TERT-HA^ and SAOS^TERT-HA^ cell lines. After the infection, cells were selected with puromycin (2μg/ml) (Tocris, USA) for at least 10 days.

### Cell fractionation and preparation of mitoplast

Mitochondria were isolated from the different cell lines using the Mitochondria/Cytosol Fractionation Kit (Abcam, Waltham, MA, USA), according to the manufacturer’s protocol but with optimized modifications to completely remove cytosolic and nuclear contamination.

Mitoplast were prepared as follow: 200μg of mitochondria were resuspended in 0.25M sucrose, 20mM Tris-HCl (pH 7.4), supplemented with 100mM CaCl_2_ and subjected to digestion with 6,000 gel units of micrococcal nuclease (New England Biolabs, Ipswich, MA, USA) and 1μg/μl digitonin (Sigma-Aldrich, Merck, Darmstadt, Germany), at room temperature for 35 minutes. Reaction was stopped with 0.5mM EDTA and mitochondria collected by centrifugation at 13,000 rpm for 2 minutes and washed twice in sucrose buffer.

### Protein lysate preparation and Western Blot

Cells or mitochondrial pellet were lysed in RIPA buffer (150 mM NaCl, 1% Triton X-100, 0.5% DOC, 0.1% SDS, 50mM Tris-HCl pH 8.0), complemented with protease inhibitors cocktail (Roche, Basel, Switzerland). Protein lysates (30μg) were loaded on an SDS-PAGE and transferred onto a polyvinylidene fluoride (PVDF) membrane (Immobilion-P, Millipore, Merck). After blocking in 3% bovine albumin serum (BSA) and 0.1% Tween, diluted in Tris-buffered saline solution (TBS), membranes were incubated with the following primary antibodies: anti-TERT (600–401-252S, Rockland, Limerick, USA), anti-SOD2 (sc-137254, Santa Cruz Biotechnology, Dallas, TX, USA), anti-Aco2 (NBP1–32781, Novus Biological), anti-LDH-A (sc-137243, Santa Cruz Biotechnology).

Finally, membranes were incubated with the appropriate HRP-conjugated secondary antibody (Bio-Rad Laboratories, Hercules, CA, USA). Proteins were visualized using Clarity Western ECL substrates (Bio-Rad). Images were acquired on ChemiDoc Imaging system (Bio-Rad) and protein levels were quantified using the Image Lab software (Bio-Rad). Experiments were repeated at least three times.

### Transmission electron microscopy (TEM) samples preparation

Samples were prepared for TEM analyses as previously described [14]. In detail, the different cell lines were centrifuged for 10 minutes (1800 rpm) in order to obtain a pellet that was then fixed in 4% glutaraldehyde, diluted in cacodylate buffer (pH 7.4), for 2 hours at 4°C. Subsequently, after three washes in the same buffer, samples were post-fixed in the dark in 1% osmium tetroxide (OsO_4_) for 20 minutes, dehydrated using an ascending ethanol series (70, 90, 100%) and finally embedded in Epon-Araldite 812 mixture resin (Sigma-Aldrich). Ultrathin sections (70 nm), obtained with a Reichert Ultracut S ultratome (Leica, Wien, Austria) and collected on gold grids (300 mesh, Sigma Aldrich). Finally, samples were counterstained with uranyl acetate and buffer citrate and then observed with a JEOL1400Plus transmission electron microscope (CRIETT center of University of Insubria - instrument code MIC01). Data were recorded with a MORADA digital camera system (Olympus, Tokyo, Japan). The total number and the size of mitochondria and the count of autophagosomes was assessed on thirty different slides for each sample (random fields of 180μm^2^) and were analyzed using the ImageJ software package (https://imagej.net/ij/, Bethesda, Maryland, USA).

### TERT localization by immunogold labeling at TEM

Samples, prepared as described above, were treated for 30 minutes with BSA blocking solution (1% Bovine Serum Albumin, 0.1% Tween in PBS), which was also used to dilute both primary and secondary antibodies. Subsequently, they were then incubated for 1 hour with rabbit polyclonal anti-TERT primary antibody (Rockland) and, after three washes with PBS, treated with goat anti-rabbit IgG(H + L)-gold conjugated secondary antibody (GE Healthcare, Amersham, UK; 10 nm particle size) for 45 minutes. In control experiments, the primary antibody was omitted. Before the observation, cells were treated for 5 minutes with 0.5% glutaraldehyde diluted in PBS, counterstained with uranyl acetate, and observed as previously described. The total number of gold particles and the related percentage was assessed on thirty different slides for each sample (random fields of 8μm^2^) and were analyzed using the ImageJ software package.

### Electron microscopy *in situ* hybridization (EM-ISH) of TERC

Cells, prepared as described above, were treated for the *in-situ* hybridization assay. Proteolytic and nucleic acids removal treatments were conducted to eliminate any possible structural proteins and DNA fragments that could impair the association between the RNA probe and its target. Slides were consecutively incubated with 20µg of Proteinase K (Thermo Fisher Scientific, Waltham, USA) and 1U of DNase I (Thermo Fisher Scientific) for 10 minutes at 37°C in a wet chamber. Afterward, a hybridization mixture was prepared containing 10µg/mL of biotin-labeled TERC probe (5’-biotin-AATGAACGGTGGAAGGCGGCA-3’), 50% formamide in saline–sodium citrate buffer (SSC). Hybridization was developed for 30 minutes at 37°C and then samples were washed three times with SSC and phosphate buffer (PBS, pH 7.4) solutions for 5 minutes. Finally, immunodetection was performed using an antibody indirect method. Samples were treated for 30 minutes with BSA blocking solution and then incubated with the monoclonal rabbit anti-biotin primary antibody for 1 hour. Following several washings in PBS, incubation with secondary mouse anti-rabbit IgG (H + L)-gold conjugate antibody (GE Healthcare; 5 nm particle size) was performed for 30 minutes. In control experiments, the primary antibody was omitted. Samples were ultimately counterstained with uranyl acetate and the precise localization of TERC inside cells and mitochondria was observed as above.

### Quantification of TERC and mRNA levels by RT-qPCR

RNA was extracted from whole cells, isolated mitochondria, or mitoplasts, with EuroGold TriFast reagent (Euroclone), according to the manufacturer’s instructions. The integrity of RNA samples was evaluated by run on 1% agarose gel electrophoresis and the concentration was quantified using Nanodrop 1000 Spectrophotometer (Thermo Fisher Scientific). RNA was reverse transcribed with the SuperScript III Reverse Transcriptase kit (Thermo Fisher Scientific), using random hexamers as primers for the reaction. Quantitative PCR was performed with AceQ Universal SYBR qPCR Master mix (Vazyme, Nanjing, PRC) using the Agilent AriaMax real-time PCR system (Agilent Technologies, Santa Clara, CA, USA), with the primers listed in S1 Table.

Relative gene expression was calculated using the 2^-ΔΔCt^ method, i.e., expression of the gene of interest relative to the reference gene (obtained by differences of threshold cycles, ΔC_T_) in a cell line was normalized to the value of the reference cell line. In the analysis of total RNA the reference gene was GAPDH, while for RNA extracted from mitochondria or mitoplast the internal mitochondrial gene MT16S was used (see S1 Table). Each experiment was conducted at least in triplicate.

### Real time quantitative–telomerase repeat amplification protocol assay (RQ-TRAP)

Telomerase activity was measured by the SYBR green RT-qPCR assay, which was conducted as previously described [14]. Briefly, the reaction was performed with protein extracts, 0.1µg of telomerase primer TS, and 0.05µg of anchored return primer ACX, in SYBR Green PCR Master Mix (Bio-Rad, USA). The reaction was performed using the Agilent AriaMax real-time PCR system (Agilent Technologies): samples were incubated for 30 min at 30°C and amplified in 40 PCR cycles with 30 sec at 95°C and 90 sec at 62°C (two step PCR). Telomerase activity was expressed relative to the telomerase positive HCT116 cells and a sample without cell lysate was used as negative control. Each sample was analyzed in triplicate in at least three independent experiments.

### Mitochondrial DNA immunoprecipitation (mIP)

For mIP, 1x10^6^ cells were subjected to cross-link with 1% formaldehyde (37%, Sigma-Aldrich) for 10 minutes at room temperature (RT). The reaction was blocked with 0.25 M glycine for 5 minutes at RT. Cells were lysed with RIPA buffer complemented with protease inhibitors (Roche) and 1 mM PMSF and homogenised with Dounce for 30 times. After incubation on ice for 30 minutes, lysates were sonicated at 60% amplitude with 10 pulses of 30 seconds, with 30 seconds rest, using a probe-type sonicator (Vibra-cell VCX130, Sonics, Newtown, CT, USA) to obtain fragments in the range between 500 bp and 1000 bp (checked by agarose gel electrophoresis). After centrifugation (5 min at 5000 rpm), the cross-linked lysate was obtained, and a small portion was stored as the input sample. Lysate was precleared on agarose beads (protein A/G PLUS-Agarose, Santa Cruz) for 1 hour at 4°C and then incubated with magnetic beads conjugated with the antibody that recognises the HA-peptide (Pierce Anti-HA Magnetic Beads, Thermo Fisher Scientific) for 2 hours at RT. At the end of the incubation, the beads were washed 5 times for 3 minutes with the following buffers: 1) 20mM Tris-HCl (pH 8.0), 150mM NaCl, 2mM EDTA, 1% Triton-X100, 0.1% SDS; 2) 20mM Tris-HCl (pH 8.0), 500mM NaCl, 2mM EDTA, 1% Triton-X100, 0.1% SDS; 3) 50mM Tris, 250mM LiCl, 2mM EDTA, 0.5% NP40, 0.5% DOC; 4) 10 mM Tris-HCl (pH 8.0), 1mM EDTA (twice). Immunoprecipitated sample was eluted from the beads with 50mM NaHCO_3_, 1% SDS for 15 minutes. After adding 0.2M NaCl, crosslinks were reversed overnight at 65°C. The day after the samples were incubated for 1 h at 45°C with 20µg of RNase A and 40µg of proteinase K, in 40mM Tris-HCl (pH 6.5), 10mM EDTA. DNA was then recovered by phenol/chloroform extraction and ethanol precipitation and subsequently analysed by PCR amplification with the primers designed specifically for different mitochondrial genes and listed in the S1 Table. The PCR reaction was performed on a thermocycler (Bio-Rad) with Taq 5X Master Mix (New England Biolabs) for 35 cycles with the following thermal cycling conditions: initial denaturation 95°C 30 sec, denaturation 95°C 15 sec, primer annealing 58°C 15 sec and elongation 68°C 40 sec. PCR products were analysed on 1.2% agarose gel electrophoresis. The gel was visualized by the ChemiDoc detection system (Bio-Rad) and analysed with ImageJ software (USA).

The immunoprecipitated DNA was further analysed by quantitative PCR (qPCR) to quantitatively determine the enrichment of two mtDNA sequences (ND1 and ND3). qPCR was performed as described in a previous paragraph, with the primer rt-ND1 and rt-ND3 (reported in S1 Table). IP fold enrichment was obtained by the 2^-ΔΔCt^ method, from the difference of threshold cycle ΔC_T_ in the IP sample relative to Input sample, in a cell line normalized to the control cell line.

Experiments were repeated at least four times.

### mtDNA replication analysis by dot-blotting

To analyse mtDNA replication 6x10^5^ cells were seeded in a plastic petri dish of 66mm. 24 hours after, cells were first treated with 240μM aphidicolin (APH; Sigma-Aldrich) for 2 hours and then with 15μM 5-bromo-2-deoxyuridine (BrdU) for 2 hours. Subsequently, cells were harvested and subjected to total DNA extraction. The cell pellet was incubated in Tail Lysis Buffer (100mM Tris-HCl, pH 8.5; 5mM EDTA, pH 8.5; 200mM NaCl; 0.2% SDS) supplemented with 30µg of Proteinase K overnight at 55°C. The following day DNA was isolated by phenol/chloroform extraction and 2-Propanol precipitation. To eliminate RNA, the DNA samples were treated with 20 µg of RNase A for 1 hour at 45°C and then subjected to ethanol precipitation.

To visualize the BrdU signal, dot blot analysis was performed. The extracted DNA was denatured in 1.5M NaOH and 1M NaCl for 10 minutes at room temperature, then diluted in 0.1X SSC; 0.125M NaOH 5 minutes on ice and subsequently blotted on a nitrocellulose membrane (Amersham Hybond -N + , Cytiva). The membrane was subjected to a cross-link with CL-508 CROSSLINKER (UVITEC, Cambridge) and after being washed with TBS-1% Tween, it was blocked with 5% non-fat milk in TBS-T for 1 hour at room temperature (RT). Subsequently the membrane was incubated with anti-BrdU antibody (Dako Denmark, monoclonal Mouse) in 1% non-fat milk in TBS-T for 2 hours at RT. After being washed with TBS-T, it was incubated with secondary anti-mouse antibody in 1% milk in TBS-T for 1 hour at RT. At the end, the membrane was washed in TBS-T. The BrdU signal was detected by chemiluminescence with Clarity Western ECL (Bio-Rad). The signal was visualized by the ChemiDoc enhanced chemiluminescence detection system (Bio-Rad) and analysed with ImageJ software. Experiments were repeated at least three times.

### mtDNA replication analysis by immunofluorescence

To analyse mtDNA replication of U2OS and U2OS^TERT-HA^ 8x10^4^ cells were seeded in a 35mm petri dish. After treatments with APH and BrdU, as described above, cells were fixed with methanol for 20 minutes and then permeabilized in PBS – 0.3%Triton X-100. DNA denaturation with 1.5M HCl for 30 minutes and then neutralization with 0.1M Sodium Tetraborate (Na_2_B_4_O_7_) were carried out. Slides were incubated with anti-BrdU antibody (Dako Denmark, monoclonal Mouse) in 1% PBS/BSA for 1 hour at 37°C. Samples were washed in PBS then incubated with secondary antibody (Invitrogen, Alexa Fluor 488 1:200 donkey anti-mouse IgG) in 1% PBS/BSA for 1 hour at 37°C. DNA was counterstained by DAPI (4’-6-diamidin-2-phenylindole) (Sigma-Aldrich) and Vectashield anti-fade (Vector Laboratories, Burlingame, CA). Slides were analysed using Axio Imager M1 microscope (Carl Zeiss, Germany) equipped with Cool Cube 1 (CCD) camera and DAPI and FITC specific filters. For each experimental point, 50 nuclei were analysed and was conducted at least in triplicate.

### mtDNA copy number

Total DNA was purified from cells using the phenol/chloroform extraction method, as described above. An amount of 5 ng of DNA was used for qPCR analysis of mitochondrial DNA using primer for the mitochondrial D-loop region and for the single copy nuclear gene β2M (sequences reported in S1 Table). The mitochondrial DNA content was determined as relative to nuclear DNA, using the formula 2^-ΔCt^, where ΔCt = Ct_D-loop_-Ct_β2M_. Experiments were repeated at least four times.

### Prediction of TERT protein-protein interactions

In order to predict the probability of interactions between TERT and proteins of the nucleoid complex and/or related to mtDNA replication/transcription, we used the CT module from the PEPPI platform (Bell et al., 2022). This neural network-based module transforms the input amino acid sequences into a fixed-length vector according to the conjoint triad method (Shen et al. 2007) and classifies the resulting vector through a neural network model, thus giving a probability of interaction. We included positive and negative controls (i.e., proteins known to interact or not with TERT), in order to test the reliability of the predictions.

### Analysis of mitochondrial mass by mitotracker green staining

To analyse the mitochondrial mass of U2OS and U2OS^TERT-HA^ 8x10^4^ cells were seeded in a 35mm petri dish. Mitochondrial mass per cell was measured using the mitochondria-specific dye MitoTracker Green (Molecular Probes). Briefly, cell medium was removed and washed once with PBS and then incubated with 200nM MitoTracker Green for 30 minutes at 37°C in the dark. After two washes with PBS, fluorescent images were acquired by Axio Imager M1 microscope (Carl Zeiss) equipped with a CCD camera. FITC (500 nm emission) filter was used to acquire images at 20X magnification.

Acquired images were analyzed with ImageJ for green fluorescence. At least 100 cells per sample were analyzed and the experiment was performed four times.

### Quantification of ATP production by Seahorse XF Real-Time ATP Rate Assay

Cells were seeded on an 8-well Seahorse XF HS Miniplate (Agilent Technologies, Santa Clara, CA) at 7200 cells/well (for U2OS cell lines) or 11000 cells/well (for HFFF2 cell lines) and incubated overnight. The day of the assay, culture media was replaced with Seahorse XF DMEM (1mM pyruvate, 2mM glutamine, 10mM glucose, pH 7.4) followed by 1 hour incubation at 37 °C, in the presence of 0% CO_2_. Oxygen consumption (OCR) and extracellular acidification rate (ECAR) was monitored by Seahorse XF HS Mini analyzer (Agilent Technologies) following the sequential injection of Oligomycin and rotenone/antimycin. The assay was performed using Seahorse XF Real-Time ATP Rate Assay kit (Agilent Technologies) following the manufacturer instructions. After data acquisition, mitochondrial (oxidative phosphorylation) and glycolytic ATP rates (pmol/min) were calculated from the measured OCR through Agilent Seahorse Analytics software. Values were subsequently normalized to total micrograms of protein, determined from lysis in RIPA buffer directly on cells in each well. Experiments were repeated four times.

### Statistical analyses

When comparing different groups of cells each against all the others, ANOVA test followed by Dunnett’s test was used. When comparison was done against a single control group, unpaired or paired Student’s t-test was used, depending on the types of data. P-values below 0.05 were considered statistically significant.

## Supporting information

S1 Fig Validation of TERT-HA transduction and evaluation of TERC expression. (A) Western blot of control and transduced U2OS and SAOS cell lines. Tubulin was used as a control protein. (B) Levels of telomerase RNA component TERC evaluated by RT-qPCR. Values are expressed as fold change relative to GAPDH expression, normalized to SK-MEL28 cell line (mean ± SEM). Statistical analysis was performed compared to U2OS. *p < 0.05, ***p < 0.001 by paired Student’s t-test. (C) Telomerase activity measured by RQ-TRAP assay in different cell lines, relative to HCT116 sample. Values are expressed as mean ± SEM. Statistical analysis was performed compared to HCT116. **p < 0.01, ***p < 0.001 by paired Student’s t-test.
(TIF)

S2 Fig TEM imaging to investigate the subcellular localization of TERT and TERC. (A) Representative micrographs of TERT immunogold on TERT-negative cell lines (U2OS and SAOS) and a negative control sample (CTRL-), which refers to SK-MEL28 incubated without secondary antibody. (B) Representative micrographs of EM-ISH with biotin-labeled TERC RNA probe for TERC-positive cell lines (HCT116, SK-MEL28, SAOS and SAOS^TERT-HA^). Mitochondria (m), nucleus (n) and TERC gold particles (black arrows) are indicated. (C) Representative micrographs of EM-ISH on TERC-negative cell lines (U2OS and U2OS^TERT-HA^); CTRL- represents a negative control and refers to SAOS^TERT-HA^ incubated with RNAse A. Scale bar: 300nm.
(TIF)

S3 Fig Western Blot analysis after anti-HA immunoprecipitation. Representative image of Western Blot of immunoprecipitation performed on whole lysates of U2OS^TERT-HA^ and U2OS cells. Input samples represent cell extracts before the immunoprecipitation and IP corresponds to immunoprecipitated samples. LDH-A was used as control protein.
(TIF)

S4 Fig TEM analysis for the quantification of mitochondrial number and size, and autophagosome count. (A) Representative electron micrographs from TEM of U2OS, U2OS^TERT-HA^, HFFF2 and HFFF2^TERT-HA^ cells. m = mitochondria. (B) TEM representative micrographs displaying auto/mitophagic vacuoles (autophagosomes and mitophagosomes, respectively) in U2OS and U2OS^TERT-HA^ cells. Black arrowheads and black arrows indicate autophagosomes and mitophagosomes, respectively. n = nucleus, m = mitochondria. Scale bar: 2 μm.
(TIF)

S5 Fig Kinetic profiles of oxygen consumption rate (OCR) measurements. (A) HFFF2 and HFFF2^TERT-HA^ cell lines; (B) U2OS and U2OS^TERT-HA^ cell lines.
(TIF)

S1 Table List of primers used in this study. (PDF)

S1 Data Numerical data underlying graphs. (XLSX)
