# Supplementary figures and images for: TERT translocation to mitochondria: Exploring its role in mitochondrial homeostasis
Source: PLoS Genet. 2025 Oct 27;21(10):e1011923. doi: 10.1371/journal.pgen.1011923 (PMC12582504; doi:10.1371/journal.pgen.1011923)

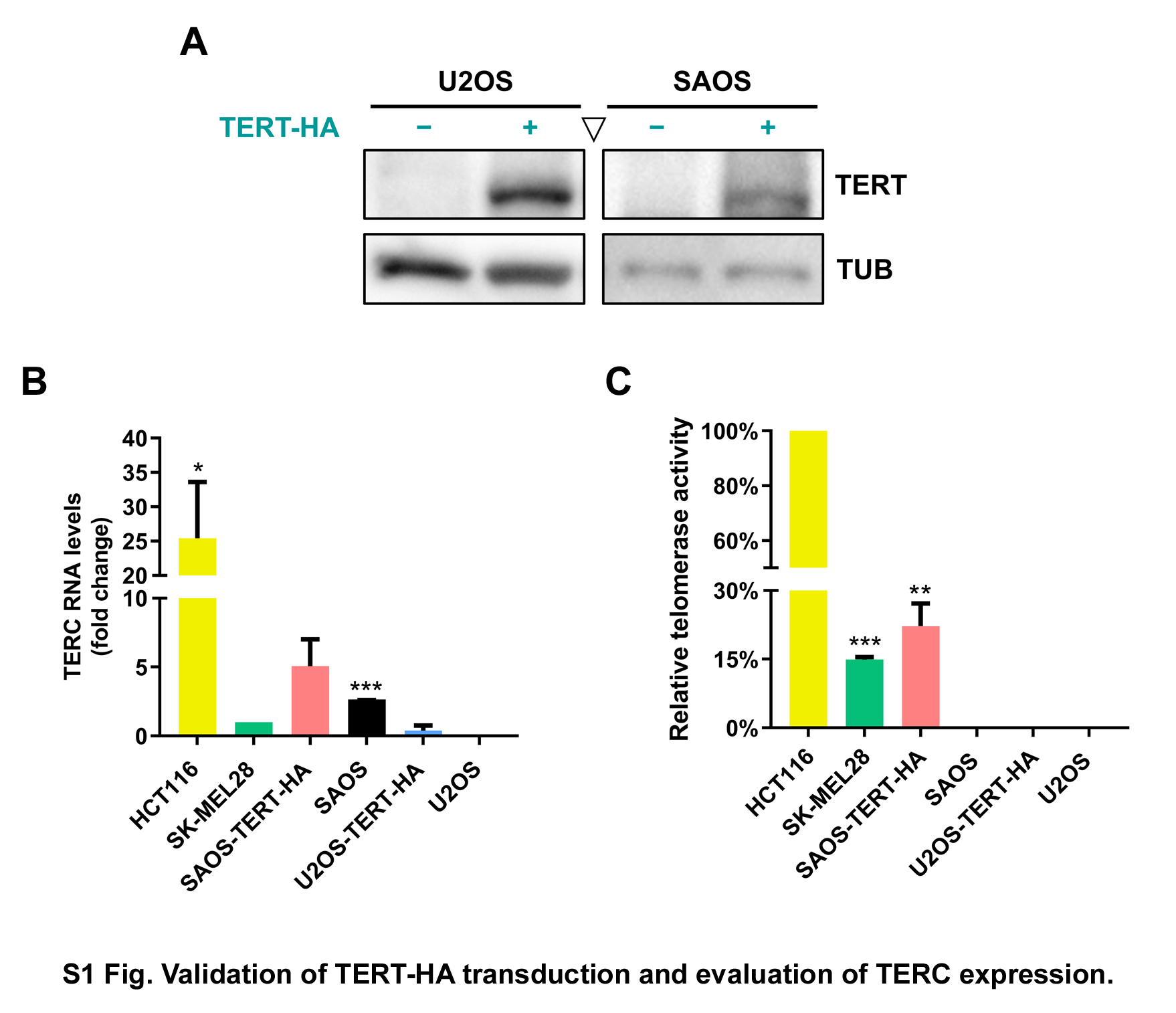

Supplement: S1 Fig — (A) Western blot of control and transduced U2OS and SAOS cell lines. Tubulin was used as a control protein. (B) Levels of telomerase RNA component TERC evaluated by RT-qPCR. Values are expressed as fold change relative to GAPDH expression, normalized to SK-MEL28 cell line (mean ± SEM). Statistical analysis was performed compared to U2OS. *p < 0.05, ***p < 0.001 by paired Student’s t-test. (C) Telomerase activity measured by RQ-TRAP assay in different cell lines, relative to HCT116 sample. Values are expressed as mean ± SEM. Statistical analysis was performed compared to HCT116. **p < 0.01, ***p < 0.001 by paired Student’s t-test. (TIF) [file pgen.1011923.s001.tif]

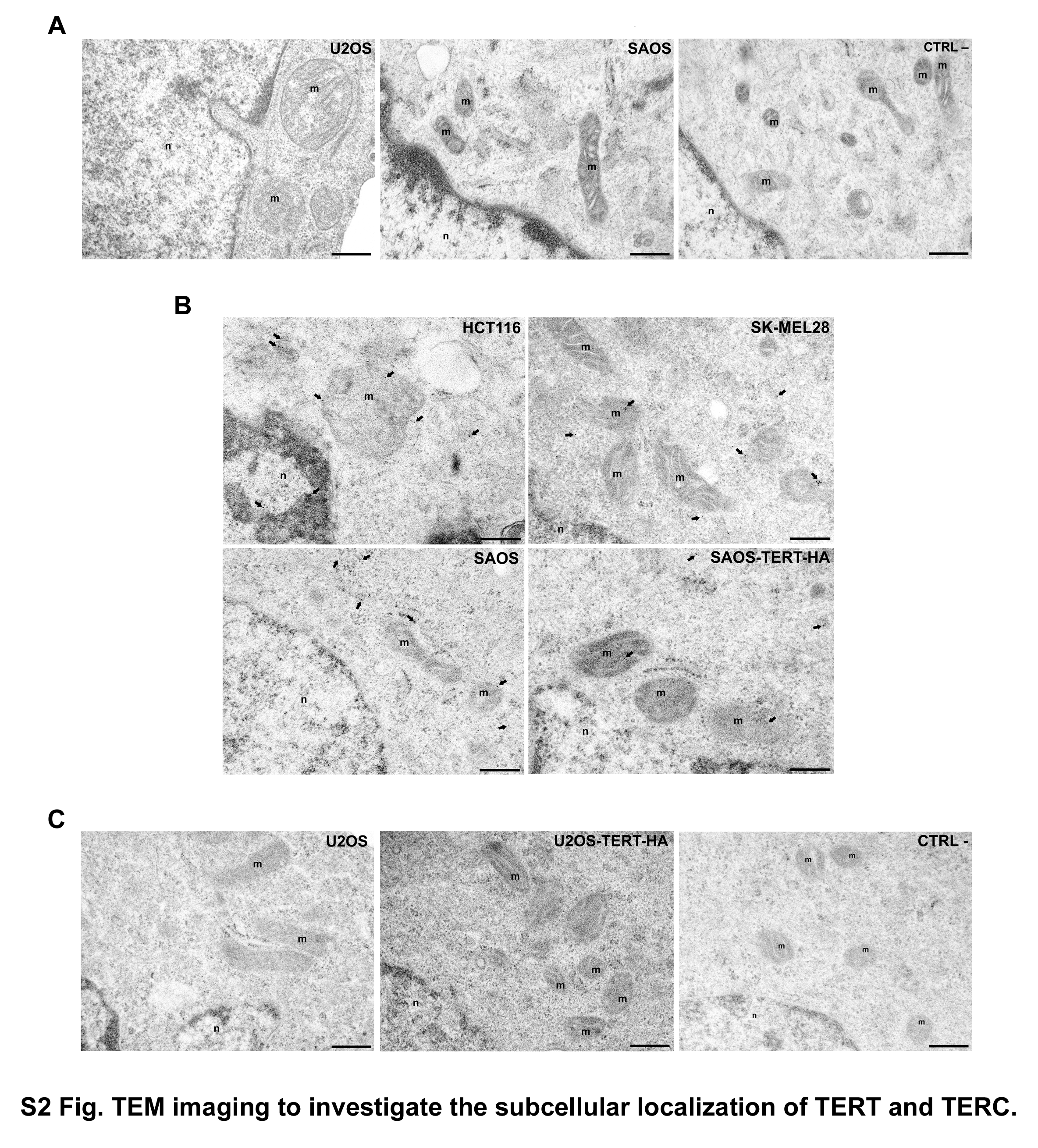

Supplement: S2 Fig — TEM imaging to investigate the subcellular localization of TERT and TERC. (A) Representative micrographs of TERT immunogold on TERT-negative cell lines (U2OS and SAOS) and a negative control sample (CTRL-), which refers to SK-MEL28 incubated without secondary antibody. (B) Representative micrographs of EM-ISH with biotin-labeled TERC RNA probe for TERC-positive cell lines (HCT116, SK-MEL28, SAOS and SAOSTERT-HA). Mitochondria (m), nucleus (n) and TERC gold particles (black arrows) are indicated. (C) Representative micrographs of EM-ISH on TERC-negative cell lines (U2OS and U2OSTERT-HA); CTRL- represents a negative control and refers to SAOSTERT-HA incubated with RNAse A. Scale bar: 300nm. (TIF) [file pgen.1011923.s002.tif]

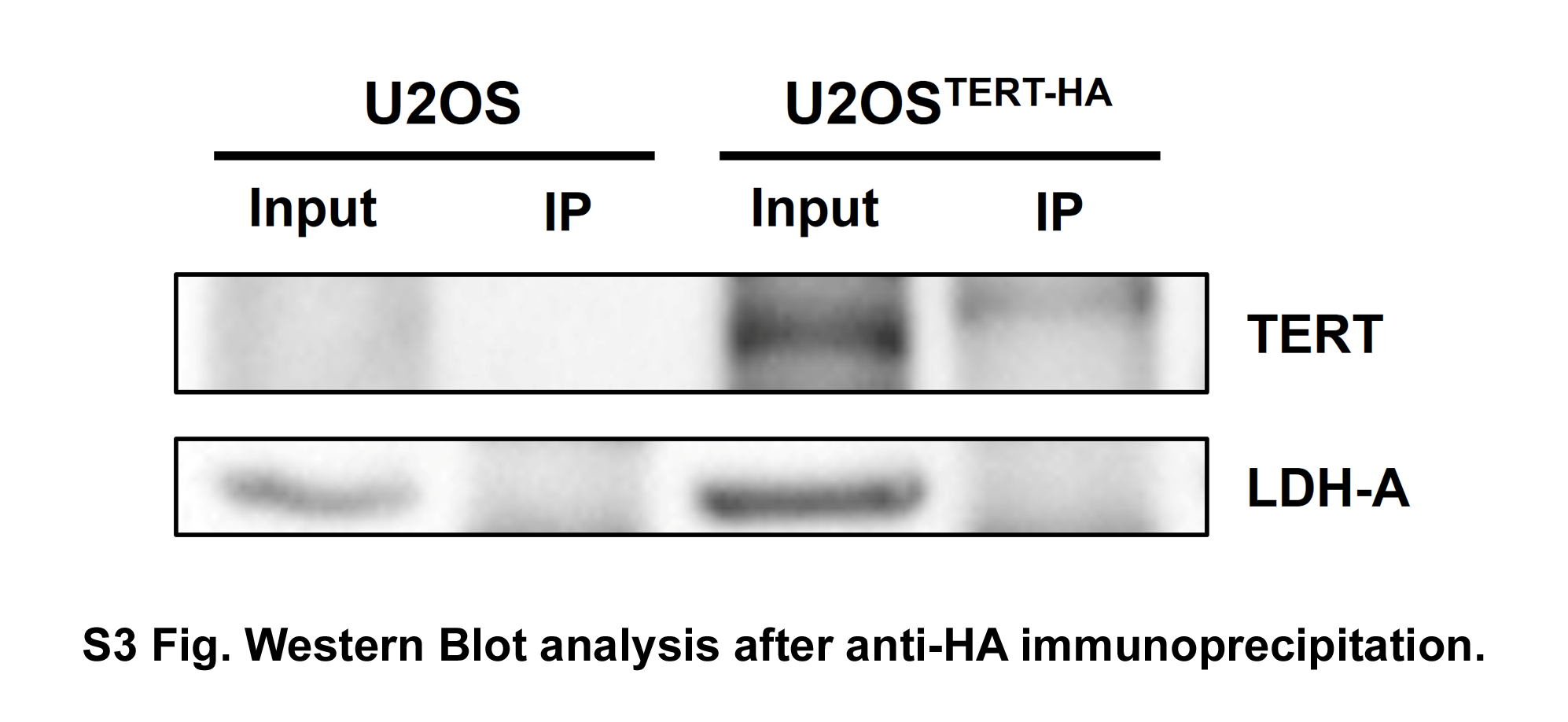

Supplement: S3 Fig — Representative image of Western Blot of immunoprecipitation performed on whole lysates of U2OSTERT-HA and U2OS cells. Input samples represent cell extracts before the immunoprecipitation and IP corresponds to immunoprecipitated samples. LDH-A was used as control protein. (TIF) [file pgen.1011923.s003.tif]

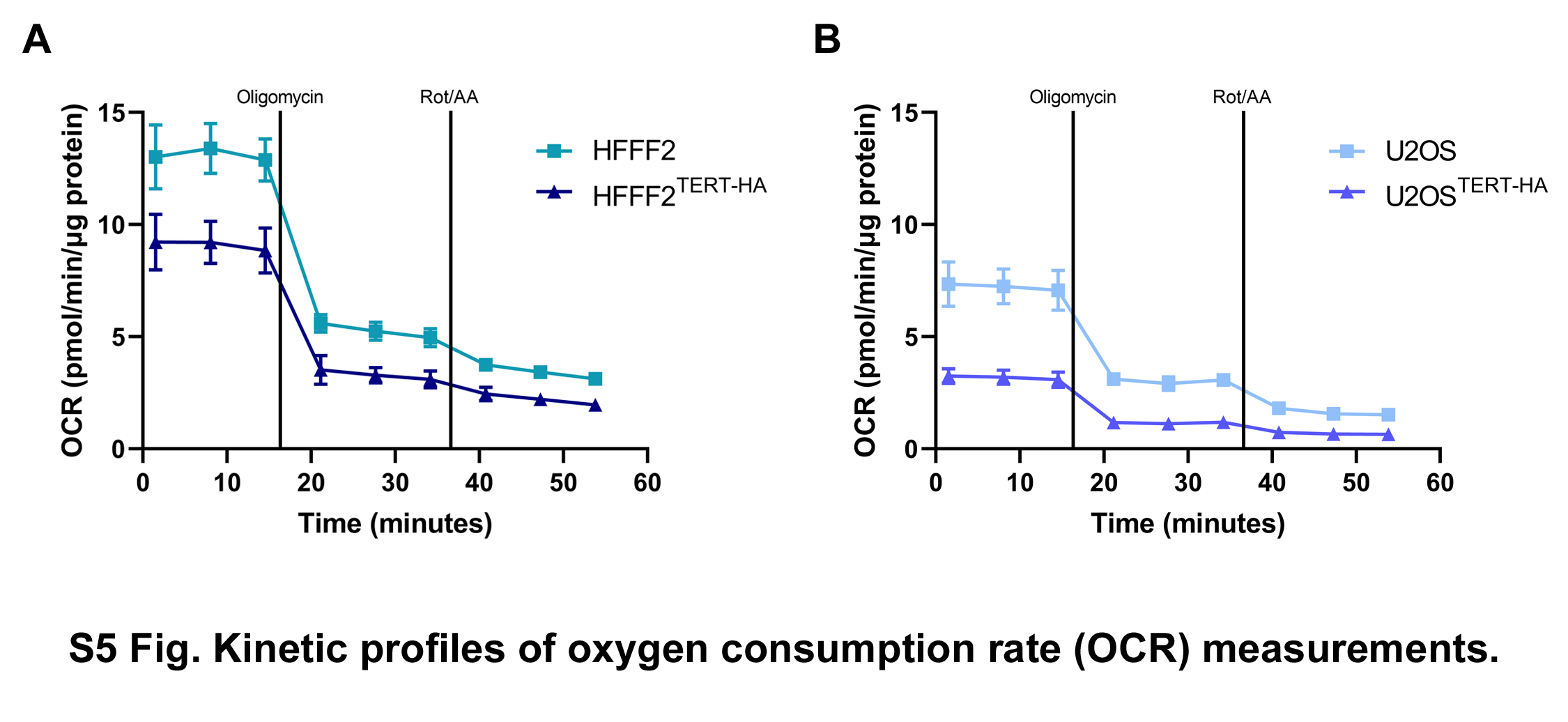

Supplement: S5 Fig — (A) HFFF2 and HFFF2TERT-HA cell lines; (B) U2OS and U2OSTERT-HA cell lines. (TIF) [file pgen.1011923.s005.tif]
