# Supplementary material for: TERT translocation to mitochondria: Exploring its role in mitochondrial homeostasis
Source: PLoS Genet. 2025 Oct 27;21(10):e1011923. doi: 10.1371/journal.pgen.1011923 (PMC12582504; doi:10.1371/journal.pgen.1011923)
Supplement: S1 Table — (PDF) [file pgen.1011923.s006.pdf]

	Forward primer (5’-3’)	Reverse primer (5’-3’)
**TERC**	CGCTGTTTTTCTCGCTGACT	GCTCTAGAATGAACGGTGGAA
**rt-ND1**	TCTCACCATCGCTCTTCTACT	AGGCTAGAGGTGGCTAGAATAA
**rt-ND3**	CCACAACTCAACGGCTACATA	AGGAGGGCAATTTCTAGATCAAA
**rt-ND5**	ACCGCACAATCCCCTATCTAGG	TTGGGTTGAGGTGATGATGGAG
**rt-COX1**	GGAGCAGGAACAGGTTGAACAG	GTTGTGATGAAATTGATGGC
**GAPDH**	AGCCACATCGCTCAGACAC	GCCCAATACGACCAAATCC
**MT16S**	CAGACCGGAGTAATCCAGGTCGG	GGAAGGCGCTTTGTGAAGTAGGC
**ND1**	CCCTAAAACCCGCCACATCT	TCAGGGGAGAGTGCGTCATA
**ND3**	GCTATTACCTTCTTATTATTTGATC	GTAGTCTAGGCCATATGTGTTGGAG
**ND5**	CCCACTACTAGGCCTCCTCC	GCGAGGGCTGTGAGTTTTAG
**COX1**	CCCTCTCTCCTACTCCTGCTCG	AGATCATTTCATATTGCTTCCGT
**D-loop**	CATCTGGTTCCTACTTCAGGG	TGAGTGGTTAATAGGGTGATAGA
**β2M**	GGATTGGTATCTGAGGCTAGTAGG	GAAATGAGGCTGGCAGAATAGG

S1 Table. List of primers used in this study.
